# How workers respond to social rewards: evidence from community health workers in Uganda

**DOI:** 10.1093/heapol/czaa162

**Published:** 2020-11-18

**Authors:** Reajul Chowdhury, Kevin McKague, Heather Krause

**Affiliations:** Agriculture and Consumer Economics, University of Illinois, Urbana – Champaign, Urbana, IL 61801, USA; Shannon School of Business, Cape Breton University, Sydney, Nova Scotia B1M 1A2, Canada; Principal Data Scientist, Datassist, Toronto, ON M4Y 3E1, Canada

**Keywords:** Community health workers, motivation, social recognition, status competition

## Abstract

This paper investigates the effect of a non-financial incentive—a competitive annual award—on community health workers’ (CHWs) performance, an issue in the public health literature that has not been explored to its potential. Combining data on a competitive social ‘Best CHW’ award with the monthly performance of 4050 CHWs across Uganda, we examined if introducing social recognition awards improved the performance of CHWs. In contrast to predominant explanations about the effect of awards on motivation, our first multilevel mixed-effect models found that an award within a branch (consisting of ∼30 CHWs) was negatively associated with the performance of the local peers of the winning CHW. Models focused on non-winning branch offices revealed two additional findings. First, a branch showed underperformance if a CHW from any of the three neighbouring branches won an award in the previous year, with average monthly performance scores dropping by 27 percentage points. Second, this negative association was seen only in the top 50th percentile of CHWs. The bottom 50th percentile of CHWs exhibited increased performance by 13 percentage points. These counter-intuitive results suggest that the negative response from high performers might be explained by their frustration of not winning the award or by emotions such as envy and jealousy generated by negative social comparisons. Our results suggest that more fine-grained examination of data pertaining to motivators for CHWs in low-income countries is needed. Motivational incentives like awards may need to be customized for higher- and lower-performing CHWs.

**KEY MESSAGES**

- Combining data on a competitive social reward intervention and monthly performance of 4050 community health workers (CHW) from Uganda, this study documents a negative association between an awarded health worker and the performance of neighbouring colleagues.
- The discretionary nature of the award, high degree of freedom of the supervisors in selecting nominees, rarity and low frequency of the award, and the social comparison costs can be potential sources of this negative association.
- Our results also showed that this association between social award and health workers’ performance varied across CHW quality tiers. Non-winning high performer CHWs exhibited underperformance when someone from a neighbouring branch wins the award, whereas the low performers CHWs reacted by improving their performance. Our results are in line with literature claiming upward-comparison induced by social awards at workplace can cause the high performers react with negative emotion like envy, and jealousy.

## Introduction

Half of the world’s population lacks access to essential health services due to financial and human resource constraints, especially in low-income countries (World Health Organization, 2017). Finding a cost-effective strategy to reach marginalized populations with basic health services has been the priority of many low- and middle-income countries, as well as the World Health Organization for decades (Perry *et al.*, 2014). Use of community health workers (CHWs) has emerged as a cost-effective approach to extend the reach of health systems to millions of the world’s most vulnerable populations (Swider, 2002; Zachariah *et al.*, 2009; Neupane *et al.*, 2018).

CHWs are volunteers or paid workers that are often used in communities beyond the reach of health facilities. CHWs are ∼70% women and are generally chosen from the communities in which they live to minimize language or cultural barriers (McKague and Harrison, 2019). They receive basic training which often includes pregnancy care, the assessment and treatment of malaria and diarrhoea and the promotion of healthy behaviours such as hygiene, immunizations, nutrition and family planning (Aitken, 2014). CHWs often go door-to-door providing primary healthcare services to the population and some also sell non-prescription medicines (e.g. contraceptives, zinc tablets, soap and period products) (Reichenbach and Shimul, 2011).

The CHW approach and its effectiveness in reaching marginalized communities with basic health services have been rigorously studied in the public health literature. Evidence shows that CHWs can be successful in low-income countries in reducing under-five child mortality by up to 25% (Perry *et al.*, 2014; Neupane *et al.*, 2018) and maternal mortality by up to 37% (Kane *et al.*, 2010). CHWs can also increase breastfeeding practice among new mothers (Hermann *et al.*, 2009). This low-cost approach to health service delivery is now a vital part of health systems in many low-income countries (Perry *et al.*, 2014). However, some studies question the sustainability of the CHW approach, pointing to two important management challenges: inconsistent delivery of services (Rowe *et al.*, 2005; Nkonki *et al.*, 2011; Singh *et al.*, 2015) and a high CHW drop-out rate (Rowe *et al.*, 2005; Brunie *et al.*, 2014; Singh *et al.*, 2015; Vareilles *et al.*, 2015). Since CHWs are not directly supervised on a daily basis, their level of service delivery can be inconsistent. The high drop-out rate of CHWs is believed to be the consequence of low motivation due to insufficient financial incentives (Nkonki *et al.*, 2011; Brunie *et al.*, 2014; Kok *et al.*, 2015), as they are not paid well or not paid at all (Perry *et al.*, 2017).

Although it is often assumed that low levels of compensation contribute to high drop-out rates among CHWs, a field experiment from Uganda found that offering financial incentives may not provide the desired or expected health outcomes in communities served by CHWs (Deserranno, 2019). In this study, the offer of greater financial incentives attracted less socially motivated people to apply to CHW jobs and resulted in high drop-out rates.

Altruistic motivation and the desire to elevate their social status in their communities have also been identified as important motivating factors for CHWs (Mlotshwa *et al.*, 2015; Kane *et al.*, 2020). Several studies have documented evidence that non-financial incentives such as moral support, appreciation and recognition from communities and organizations improve the performance of CHWs (Maes and Kalofonos, 2013; Druetz *et al.*, 2015; Vareilles *et al.*, 2017). However, quantitative empirical evidence on how non-financial social incentives affect the motivation and performance of CHWs is limited in the existing literature (Brunie *et al.*, 2014; Kok *et al.*, 2015, 2017; Vareilles *et al.*, 2015; Rachlis *et al.*, 2016; de Vries and Pool, 2017).

Non-financial incentives such as social recognition and awards have been researched in other fields, including labour economics and organizational behaviour, where there is evidence that giving social rewards to workers has a positive effect on both the recognized workers and their co-workers (Fehr and Gächter, 2000; Charness and Rabin, 2002; Beersma *et al.*, 2003; Rege and Telle, 2003; Bewley, 2007; Segal and Sobel, 2007). Beersma *et al.* (2003) showed evidence from an experimental study that a competitive non-financial reward structure (publicly appreciating and rewarding the best performer among the workers) enhanced status competition among workers and improved the performance of workers in terms of speed (Beersma *et al.*, 2003). A social reward such as a ‘Best CHW Award’ involves providing recognition to individuals for their good performance and celebrating their success in public. Social rewards are distinguished from other forms of rewards by the feature of publicity (Gallus and Frey, 2017). Public recognition of an award elevates the status of the recipients and has been shown to motivate the performance of co-workers and colleagues (Fehr and Gächter, 2000; Beersma *et al.*, 2010; Ager *et al.*, 2016).

Given the positive outcomes of non-financial social rewards in other fields, and building on models proposed by Besley and Ghatak (2008), our study set out to consider whether these outcomes are transferrable to the unique situation of CHWs in Uganda working for Building Resource Across Communities (BRAC), a large non-governmental organization.

## Intervention and data

**Figure 1:**
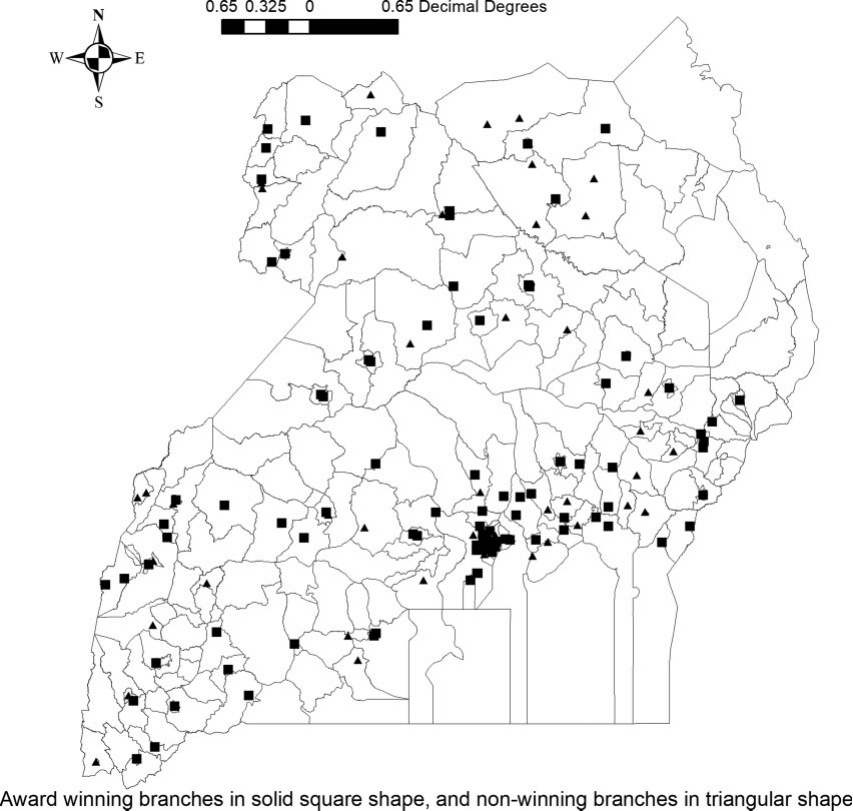
BRAC’s CHW branch offices in Uganda.

We used data from 4050 CHWs in BRAC Uganda to investigate whether introducing social rewards in the form of ‘Best CHW Awards’ enhanced status competition and improved performance of CHWs. In Uganda, CHWs are managed through 134 branch offices across the country (Figure 1) with ∼30 CHWs in each branch. The branch offices are where CHWs are recruited, trained, supervised and given supplies. BRAC recruits only female volunteer who have at least a primary level of education and have no children under-5 years of age at home. Each CHW is expected to serve ∼100 households.

### Intervention

In 2015, BRAC Uganda launched a competitive reward programme for its CHWs in the form of a ‘Best CHW Award’ that gave social recognition awards to the best-performing CHWs. At the end of each year, branch offices supplied the country headquarters of BRAC with a list of nominations of their best-performing CHWs. Nominations were based on the qualitative criteria of whether the CHW was hardworking, whether the CHW was well supported in the community and whether the CHW had been regularly attending monthly refresher trainings. The Monitoring and Evaluation (M&E) Department at BRAC headquarters then followed-up each nomination to verify the performance information. The country headquarters finalized the list of winners based on this follow-up. Award recipients received a certificate and gift items worth approximately US$100. The names of the winners and their corresponding branch offices were then shared with all CHWs and health programme staff in the following month’s refresher training meetings.

Starting in 2015, the award was given annually for 3 years. In 2015, the award was given to 46 CHWs; in 2016, the award was given to 43 CHWs; and in 2017, the award was given to 46 CHWs. In total, only 3% of the 4050 CHWs received an award, and none of the winners were awarded more than once in the 3-year period. Over the three intervention years, 88 of 134 branches (66%) had CHWs winning an award at least once. Among these 88 branch offices, 48 branches had a winner in only one year, 34 had a winner in two of the years and 6 branches had winners in each of the 3 years (Supplementary Table S1).

### Data

Our data consisted of information on the monthly activities and performance of CHWs for 21 months from October 2016 to June 2018. Table 1 presents basic descriptive statistics of the performance indicators.

**Table 1:** Descriptive statistics of major performance indicators

CHW performance indicators	Obs	Mean	SD	Min	Max
Household visits					
Number of months the CHW was active (out of 21)	85 050	13	3.81	0	21
Required follow-ups	85 050	3	5.70	0	128
On-time follow-ups made	85 050	2	4.39	0	105
Number of families registered	85 050	5	11.62	0	210
Family surveys	85 050	22	36.78	0	458
Assessment					
Number of pregnancies registered	85 050	2	2	0	103
Total health prenatal care visits	85 050	0.56	1.29	0	55
All first prenatal care visit	85 050	0.61	1.37	0	55
Assessed any patient	85 050	11	14	0	257
Number of U1 children assessed	85 050	2	3	0	80
Number of U5 children assessed	85 050	9	12	0	184
Treatment services					
Number of U1 children treated	85 050	2	3	0	76
Treatment U1: malaria	85 050	0.94	1.51	0	34
Treatment U1: diarrhoea	85 050	0.79	1.29	0	28
Treatment U1: pneumonia	85 050	0.42	0.87	0	26
Number of U5 children treated	85 050	9	11	0	306
Treatment U5: malaria	85 050	4	5	0	155
Treatment U5: diarrhoea	85 050	3	4	0	151
Treatment U5: pneumonia	85 050	2	3	0	100
Malarial all ages	85 050	5	6	0	151

Most of the performance indicators reported in Table 1 have a wide variation with high standard deviations. While there is a chance that CHWs over-reported their performance in some cases, the unusual outperformance in some months can also be explained by the recruitment of new CHWs in those months. In their first few months, newly recruited CHWs usually survey all households in their catchment areas and identify all cases of pregnancy, infant children and other patients. Though it is more likely that the unusual outperformance in some months is due to the high efforts that CHWs have to assert at the beginning of their job, we cannot claim this with certainty, as we do not have records of when a CHW drops out and get replaced by a new CHW. The 0 minimum values in the indicators suggest that there were CHWs who either dropped out or were inactive (did not work) in a particular month. Since being a CHW is not a regular salaried job and their activities are not directly monitored in the field, it is not uncommon for CHWs to become inactive in some months. Branch office supervisors are meant to terminate CHWs who remain inactive for 2 months, but they prefer to avoid such termination because it is often difficult to find new eligible candidates from their communities.

Table 2 gives a timeline of the intervention and data availability. Though the award was launched in December 2015, we could obtain the performance data only for months starting from October 2016. In October 2016, BRAC integrated mobile phone technology in its health programme, and CHWs were provided with smartphones and were required to report their activities through a mobile application. The monthly performance data for each CHW was then generated by aggregating these day-to-day activity reports. Prior to October 2016, reports were generated manually at the end of month when CHWs came to branch offices to receive refresher training. For this study, we did not have the resources to digitize pre-2016 handwritten monthly data for the 4050. Considering these challenges in our data, we designed our empirical strategy with multilevel mixed-effect models that take the hierarchical structure of the data into consideration and control for unobserved heterogeneity at different levels (see Methods and empirical models section for detail).

**Table 2:** Timeline of intervention and data availability

2015		2016		2017		2018
November to December	January to September	October	November to December	January to October	November to December	January to June
46 awards provided		Mobile reporting launched	43 awards provided		46 awards provided	

*Note*: Monthly performance data were not available in 2015 or from January to September in 2016 (shown in grey).

Despite the above-mentioned shortcomings in our data, there are several reasons that make BRAC’s CHW programme a good setting to examine the relationship between social incentives and CHW’s performance. First, the catchment area of each CHW is well defined and does not overlap with that of other CHWs, which controls for any task complementarities among them at the field level. Next, the remuneration package is identical for all CHWs regardless of their experience and educational qualifications, controlling for any externalities arising from remuneration. As the CHW job is voluntary in nature and day-to-day CHW activities are not monitored by branch managers, the externalities arising from supervision quality is expected to be limited.

### Outcome variable: performance index

Of the 21 performance indicators, selecting one or a few as the primary outcomes of interest would ignore the multi-dimensionality of CHW performance. Measuring the effect of the awards on some particular performance indicators would make sense if the nominees were selected based on clearly defined measurable indicators (known as confirmatory rewards); however, the selection of the award recipients was made following a subjective process. Considering these challenges, we decided to generate a performance index value for each CHW and use the index as the primary outcome of interest. The idea comes from the latent variable approach, which assumes that different dimensions of CHW performance cannot be directly observed but can be represented by an index value that partially explains the variation in the observed performance indicators (Zeller *et al.*, 2006; Williams *et al.*, 2010). A latent variable approach is used widely in development economics to measure household welfare (e.g. poverty indices) (see Zeller *et al.*, 2006; Morel and Chowdhury, 2015). The two most widely used models of latent variable approach are the principal component analysis and factor analysis (FA) (Zeller *et al.*, 2006; Krishnakumar and Nagar, 2008; Williams *et al.*, 2010). In this paper, we used FA to construct the performance index value.

To improve the explanatory power of the performance index, some indicators have been filtered out from the FA. First, all but one indicator representing a particular aspect have been dropped because having many variables of a particular aspect would bias the index. For example, the variables *U-1 children treated for malaria*, *U-1 children treated for diarrhoea* and *U-1 children treated for pneumonia* were dropped from the analysis because these are closely correlated with the variable *number of U-1 children treated*. Second, following the standard procedure in the literature, the selected variables have been log transformed before being used in the FA. Therefore, any variable presenting the ratio of two other variables have been excluded. Third, dichotomous response variables such as *whether organized any community event* have not been included in the FA model, as the model requires continuous data. The final FA model includes a total of 12 variables covering different aspects of CHW performance.

We used maximum likelihood estimation to extract the number of latent factors from the data. Supplementary Table S2 in the Supplementary data shows the indicators selected in the ﬁnal model and their respective factor loadings. Factor loadings show the degree and sign of the correlation between the factors and indicators. The coefﬁcients or loadings in Factor 1 appear with expected signs in relation to CHW performance and are treated as the relative performance index. Factor 2 does not seem to consistently capture variance related to performance, as some variable loadings have unexpected signs and all but two of them have insigniﬁcant values. Using the loadings of Factor 1, we estimated the factor scores, or performance index, for each observation of monthly CHW performance.

## Methods and empirical models

Given the non-ignorable clustering and lack of independence across our observations, we recognized the need for more complex empirical models that would take the hierarchical structure of the data into consideration and control for unobserved heterogeneity at different cluster levels. Multilevel mixed-effects models—also known as hierarchical linear models and random-effects models—offered solutions to these problems (Snijders and Bosker, 2011; Gelman, 2006; Bell and Jones, 2015). We used a multilevel mixed-effect model to estimate the influence of social recognition on CHW performance. In the absence of data on CHWs and branch-level characteristics, multilevel mixed-effect method allowed us to explicitly model externalities arising from unobserved variables at the branch and CHW levels. By explicitly modelling for such externalities, the multilevel mixed-effect method separated the effect of unobserved externalities from the estimated coefficients of interest (Steele, 2008; Snijders and Bosker, 2011; Mathieu *et al.*, 2012; Aguinis *et al.*, 2013). Multilevel modelling also allowed us to investigate the nature of between-cluster variability (Gelman, 2006; Gelman and Hill, 2006; Bell and Jones, 2015), or—whether monthly performance observations varied across branch offices and CHWs and whether the variation between branch offices differed by quality of CHW.

We began by examining how performance of a CHW changed if someone from her branch won the award in the previous year. We fit a three-level hierarchical model where the monthly performance scores (level 1) are nested within CHWs (level 2) and CHWs are nested within branch offices (level 3). In our fitted model, we allowed the intercept to vary at both branch and CHW levels and let the coefficient of our variable of interest vary across branch level only. This was the best-performing model found from an exercise where several models were fitted letting the intercept and slope vary at either or both of the CHW and branch levels. Our preferred estimated model took the following form:

(1) $${Y_{tij}} = {\gamma _{000}} + {\text{}}{\beta _1}Branch{\,\,\text{}}Siz{e_j} + {\,\,\text{}}{\beta _2}\,\,Awarded{\,\,\text{}}Last{\,\,\text{}}Yea{r_j} + {v_j}{\,\,\text{}}Awarded{\,\,\text{}}Last{\,\,\text{}}Yea{r_j} + {v_j} + {u_{ij}} + {\,\,\text{}}{e_{tij{\,\,\text{}},}}$$

where:

${Y_{t,i,j}} = {\text{Performance index score of a CHW}}\,i\,{\text{from branch}}\,j\,{\text{in month}}\,t;$

${\gamma _{000}} = {\text{Grand mean of performance scores across all months}},{\mkern 1mu} {\mkern 1mu} {\text{CHWs}},{\text{and branches}};$

$Branch\,Siz{e_j} = {\text{Number of CHWs serving in the same branch}}{\mkern 1mu} j;$

$Awarded\,Last\,Yea{r_j} = I{\text{f any CHW from the same branch received an award in the last year}};$

${v_j} = {\text{Residual coefficient for performance score for branch}}\,j;$

${u_{ij}} = {\text{Residual coefficient for performance score for CHW}}\,i\,{\text{in branch}}\,j;$

${e_{tij}} = {\text{Residual coefficient for performance score for month t of CHW}}\,i\,{\text{in branch}}\,j$

We took the performance index score estimated through FA as the outcome variable. In estimating this model, we excluded the observations of the award-winning CHWs. We also made an assumption that giving a social reward to a CHW affects the performance of her colleagues in the immediate next year only.

Using the same multilevel modelling approach, we examined whether the effect of social recognition varied by CHW performance quality. As a measure of the performance quality, we took the percentile rank of CHWs based on their relative position in the distribution of the previous year’s average performance index scores. While constructing the quality measures based on the previous year’s performance scores instead of the current year’s performance scores implies losing observations and reducing power, this would make the possible endogeneity problem less severe given that the relative performance is also an outcome in our model. The estimated model was:

(2) $$\begin{gathered}{Y_{tij}} = {\gamma _{000}} + {\beta _1}Branch\,Siz{e_j} + {\beta _2}Awarded\,Last\,Yea{r_j} + {\beta _3}\,Percentil{e_{i,j}} + {\beta _4}\,Percentil{e_{ij}} \\ \times Awarded\,Last\,Yea{r_j} + {v_j}\,Percentil{e_{ij}} \\
\times Awarded\,Last\,Yea{r_j} + {v_j} + {u_{ij}} + {e_{tij}}\end{gathered}$$

In Model 2, the *percentile_ij_* is a dummy indicating the percentile bracket (top 50% i.e. 50–100th percentile, bottom 50% and bottom 20%) to which a CHW belongs based on the distribution of the average performance indicator values in the previous year. The coefficient of the interaction between the *n*th percentile rank of a CHW and the presence of an award-winning colleague (β_4_) is the coefficient of interest. The β_4_ measures how the performance of a CHW from the *n*th percentile changes if a colleague received the award in the previous year.

Finally, in the later part of our analysis, we used a more restricted sample, focusing on only the non-winner branches and examining how CHW performance from those branches changed if someone from their neighbouring three branches won the award. Restricting our focus on non-winner branches allowed us to control for externalities arising from the quality of supervision and training that might make the winner branches systematically different from the non-winner branches.

## Results

### Effect on immediate colleagues in next year

Table 3 presents the results estimated from the model specified in Equation (1). The estimate shows a non-significant negative association between the social award and CHW performance: having an immediate colleague awarded in the previous year is associated with a reduction of 4.9 percentage points on the performance index. The estimate also shows that branch size, which indicates the number of CHWs in the same branch, had a positive but statistically insignificant association with the performance scores. The likelihood ratio (LR) test comparing this model to one without clustering was highly significant supporting the choice of the fitted model. An important takeaway from the results is that the variance of the random effect (slope) of the *Awarded Last Year* variable alone accounts for a 39% variance in performance score.^1^ This further confirms our model’s assumption that the effect of having an award-winning colleague on performance of other CHWs varies significantly across branch offices.

**Table 3:** Effects on CHW peers from the same branch office

	Random intercept and slope at branch level and random intercept at CHW level
	(1)
Treatment: award-winning colleague	−0.049 [−0.225 0.126]
	(0.089)
Branch size	0.001 [−0.004 0.006]
	(0.003)
Constant	−0.089 [−0.258 0.079]
	(0.086)
ICC	
Branch	0.174 [0.141 0.214]
CHWs within branch	0.259 [0.228 0.294]
Additional information:	
*N*	85 050
LR test: compared with pooled OLS (chi)	14844***

*Note*: Performance index score is the dependent variable. 95% confidence intervals are shown in brackets and standard errors are shown in parentheses. *** denotes statistical significance of coefficients at the 1% confidence level.

As mentioned earlier, we obtained this preferred model of Equation (1) from an exercise of fitting several two-level and three-level hierarchical models with intercept and slope varied at either or both of the CHW and branch levels. The results from these estimated models are presented in Supplementary Table S3 in the Supplementary data. To complement our multilevel modelling estimates, we also fitted a fixed-effect model and performed the Hausman test to see if the assumption of non-correlation between the error terms and the covariates hold or not. The results of the fixed-effect estimations are presented in Supplementary Table S4 in the Supplementary data. Results of the Hausman test shows non-correlation between the covariates and the error terms supporting the random-effect assumption of our empirical model.

### Effects of award by CHW quality

In Table 4, we present the results estimated following the empirical model specified in Equation (2). The purpose was to investigate if the effect of the social recognition award varied across CHWs by their quality. In estimating the model, the *CHW’s nth percentile* variable was generated by taking the different percentile cut-offs of the average performance index score in the previous year. The variable is a dummy that takes the value of 1 if a CHW belonged to the *n*th percentile rank of the average performance indicator in the previous year. The percentile ranks are indicated in the column headings. For example, in Column (1) the *CHW’s nth percentile* variable takes a value of 1 if a CHW’s average performance in last year belonged to the top 50th percentile, otherwise it takes 0.

**Table 4:** Effects on the peers of different quality from the same branches

	Random intercept and slope at branch level and random intercept at CHW level		
	Top 50 percentile	Last 50 percentile	Last 20 percentile
	(1)	(2)	(3)
Treatment: Award-winning colleague	0.013 [−0.014 0.039]	0.102 [0.074 0.130]	0.035 [0.013 0.056]
	(0.013)	(0.014)***	(0.011)^***^
Branch size	0.001 [−0.001 0.004]	0.001 [−0.003 0.003]	−0.001 [−0.003 0.002]
	(0.001)	(0.001)	(0.001)
CHW’s n^th^ percentile	0.391 [0.367 0.415]	−0.369 [−0.395 −0.345]	−0.569 [−0.599 −0.539]
	(0.012)^***^	(0.013)^***^	(0.015)^***^
Treatment × CHW’s *n*th percentile	0.052 [−0.011 0.115]	−0.057 [−0.179 0.066]	0.094 [−0.051 0.239]
	(0.032)	(0.063)	(0.074)
Constant	−0.048 [−0.134 0.039]	0.365 [0.255 0.475]	0.339 [0.252 0.427]
	(0.044)^***^	(0.056)^***^	(0.045)^***^
ICC			
Branch level	0.054	0.087	0.057
	[0.042 0.069]	[0.069 0.111]	[0.044 0.074]
CHW within branch	0.148	0.188	0.145
	[0.135 0.162]	[0.169 0.209]	[0.131 0.160]
Additional information			
*N*	72 900	72 900	72 900
LR test: compared with pooled OLS	6576.31^***^	8073.24^***^	5527.30^***^

*Note*: Performance index score is the dependent variable. 95% confidence intervals are shown in brackets and standard errors are shown in parentheses. *** denotes statistical significance of coefficients at the 1% confidence level.

Table 4 shows that the interaction term between the *Awarded Last Year* variable and the *n*th percentile rank (top 50th, bottom 50th and bottom 20th) of the performance indicator was found to be trivial in all of the estimated models, suggesting there were no important differences between the subgroups. However, this relationship is quite heterogenous across the CHW and branch levels, as suggested by the random-effect parameters, variances of the intercepts and coefficient slope, and the corresponding likelihood ratio test statistics of the models.

### Effects on CHW peers in the neighbouring branches

In Tables 3 and 4, we presented findings from models focusing on immediate peers and examined how their performances changed in the presence of an award-winning CHW in the same branch. These models did not control for any correlated shocks that CHWs might have faced within a branch. Correlated shocks might have come from having the same supervisor at the branch level, receiving training from the same trainer, or serving in similar types of communities. This means that performance index scores of CHWs from award-winning branch offices might have been systematically different from that of non-winning branches. Furthermore, the inclusion of observations from winning branches in the sample while measuring the spill-over effect of the social recognition award may have diluted this effect. To control for this threat, we re-estimated the models excluding the observations from the branches having at least one CHW who received the award in the previous year.

We redefined our treatment variable of *Awarded Last Year_j_* as an indicator variable that takes a value of 1 if any CHW within the nearest three neighbouring branches received the award in the previous year. For example, for any of the months in year 2017, this dummy variable took the value of 1 if and only if any CHW within the nearest three branches received the award in December 2016. As with the previous model, we assumed that the social recognition award affected the performance of CHWs only in the preceding year.

The results are presented in Table 5. Estimates revealed a significant negative association between the variable *Awarded Last Year* and the monthly performance scores of CHWs. Presence of an award-winning CHW among the nearest three neighbouring branches was associated with a 27 percentage points decrease in the average performance score of a non-winner branch, which is significant at the 1% level. The likelihood ratio test statistics (χ^2^ = 4258.47 and *P* < 0000) comparing this model with the ordinary least squares (OLS) model showed that the current model substantially better fit the data.

**Table 5:** Effects on the peers from the neighbouring branches

	Random intercept and slope at branch level and random intercept at CHW level
	(1)
Treatment: a winner within the nearest three branches	−0.270 [−0.451 −0.089]
	(0.092)^***^
Branch size	−0.004 [−0.012 0.003]
	(0.004)
Constant	0.177 [−0.059 0.413]
	(0.121)
ICC	
Branch level	0.137
	[0.092 0.199]
CHW within branch	0.216
	[0.171 0.269]
Additional information	
*N*	26 922
LR test: compared with pooled OLS	4258.47^***^

*Note*: Performance index score is the dependent variable. 95% confidence intervals are shown in brackets and standard errors are shown in parentheses. *** denotes statistical significance of coefficients at the 1% confidence level.

Table 6 follows models similar to those in Table 4. The results show significant heterogeneity across the three groups of CHWs. The top 50th percentile group showed underperformance by 20 percentage points (*P* < 0.01) while the bottom half and bottom 20th percentile groups showed outperformance by 13 percentage points (*P* < 0.05) and 17 percentage points (*P* < 0.05), respectively. The estimates implied that CHWs from the top 50th percentile group underperformed by 20 percentage points when a CHW from any of the nearest neighbouring three branches received the award. On the other hand, CHWs from the bottom half showed an outperformance in response to the intervention. For the bottom 20th percentile group, the magnitude of outperformance was even larger.

**Table 6:** Effects on the peers of different quality from the neighbouring branches

	Random intercept and slope at branch level and random intercept at CHW level		
	Top 50 percentile	Last 50 percentile	Last 20 percentile
	(1)	(2)	(3)
Treatment: a winner within the nearest three branches	0.274 [0.231 0.317]	0.008 [−0.033 0.049]	0.090 [0.059 0.122]
	(0.022) ^***^	(0.021)^***^	(0.016)^***^
Branch size	0.002 [−0.002 0.006]	0.004 [−0.001 0.009]	0.000 [−0.004 0.004]
	(0.002)	(0.003)	(0.002)
CHW’s *n*th percentile	0.466 [0.415 0.517]	−0.382 [−0.434 −0.331]	−0.612 [−0.666 −0.558]
	(0.026)^***^	(0.026)^***^	(0.028)^***^
Treatment × CHW’s *n*th percentile	−0.199 [−0.291 −0.107]	0.131 [−0.011 0.273]	0.165 [0.007 0.323]
	(0.047)^***^	(0.073)^**^	(0.081)^**^
Cons	−0.227 [−0.363 −0.091]	0.219 [0.049 0.391]	0.239 [0.113 0.367]
	(0.069)^***^	(0.087)^***^	(0.065)^***^
ICC			
Branch level	0.054 [0.034 0.082]	0.082 [0.053 0.124]	0.048 [0.031 0.074]
CHW within branch	0.142 [0.119 0.167]	0.172 [0.142 0.208]	0.120 [0.099 0.144]
Additional information			
*N*	23 076	23 076	23 076
LR test: compared with pooled OLS	1934.38^***^	2225.05^***^	945.94^***^

*Note*: Performance index score is the dependent variable. 95% confidence intervals are shown in brackets and standard errors are shown in parentheses. ** and *** denote statistical significance of coefficients at the 5% and 1%, respectively, confidence level.

## Discussion

In this paper, we examined the relationship between social rewards and CHW performance using data from 4050 CHWs organized within 134 branch offices of BRAC Uganda, a non-governmental organization. In 2015, BRAC introduced an annual competitive award for the best-performing CHWs. Between 2015 and 2018, a total of 135 CHWs from 88 branch offices won the award. We designed an empirical strategy with multilevel mixed-effect models that allowed us to control for unobserved heterogeneity at the branch and CHW levels.

Our analysis began by looking at how CHWs reacted when a colleague from the same branch received the award in the previous year. The results showed a negative but statistically non-significant association between the award and CHW performance. However, the estimations showed that CHW-level and branch-level factors accounted for a substantial amount of variation in performance scores. The variation in performances across branch offices can be explained by differences among branch offices in terms of geographical location, quality of field supervisors and other characteristics, such as population size, infrastructure and the presence of health facilities in the community. The variation in performances across CHWs can be attributed to differences in individual factors, such as age, education, years of experience, marital status and number of children. We also found that the spill-over effect of the award significantly varied among branches but not among CHWs.

Our estimates from a restricted sample controlling for correlated shocks at branch level found mixed effects. We found a significant negative association between CHW performance and the awards: having an award-winning CHW among the nearest three neighbouring branches was associated with a 27 percentage points decrease in the average performance score of a non-winner branch.

One possible source of this negative association might be the design of the award itself. The nominations for the award were based on qualitative dimensions which gave the branch supervisors a high degree of freedom to decide upon whom the awards are bestowed. Gallus and Frey (2017) defined such awards as discretionary awards. While discretionary awards allow the supervisors to signal their intent and desired quality, such awards are also sensitive to issues of lack of objectivity. Discretionary awards can be perceived as a signal of favouritism and can deteriorate the reputation of both the supervisors and the award winners if the supervisors are not seen to invest enough time and effort in the selection of the candidates and the winners (Gallus and Frey, 2017). The discretionary nature of the awards might lead CHWs to believe that those selected by the branch supervisors were selected because of personal connections with the supervisors. Therefore, the awards might have been considered unfair, inducing a negative spill-over on CHW performance. Similarly, a review of literature on factors affecting CHW performance found that recognition by the organization improves CHW performance when the recognition is believed to be based on fairness and equity (Vareilles et al., 2017).

Another source of this negative effect might come from the non-recipients perceiving the award as a signal of them not being meritorious—a phenomenon referred to as ‘social comparison cost’ in the literature (Harlow and Cantor, 1995; Exline and Lobel, 1997; Exline *et al.*, 2004; Clark *et al.*, 2010). Giving an award to a selected group of employees involves the risk of offending the non-recipients, particularly in small and homogenous group of employees where interpersonal comparisons are dominant (Frey, 2007; Gallus and Frey, 2017). Non-recipients often respond to such a workplace award with feelings of dejection or inferiority (Salovey and Rodin, 1984; Exline *et al.*, 2004). This negative emotional response by the non-recipients may result in reduced efforts, increased jealousy and even sabotage (Charness *et al.*, 2014; Gallus and Frey, 2017).

The rarity of the award might also have induced the overall negative performance (only 3% of CHWs received the award). The low frequency of the award might have made the CHWs feel they had a low chance of winning it, which might have demotivated them and reduced their performance (Carnahan *et al.*, 2010). A logical extrapolation of this point implies that high-performing workers have a higher chance of winning the award and are more likely to be positively affected by the award compared with their low-performing colleagues. This hypothesis is also suggested by several studies arguing that high performers respond positively to social awards by identifying themselves with the award winners (Ybema and Buunk, 1995; Collins, 1996; Hoyt, 2013) or seeing the winners as a source of inspiration (Ybema and Buunk, 1995; Exline and Lobel, 1997). However, our findings suggest the opposite. Our multilevel modelling estimates on the CHWs from the non-winning branches found that the presence of an award-winning CHW in the neighbouring three branches was correlated with a significant drop in performance scores of the top 50th percentile of CHWs. On the other hand, the bottom 50th percentile CHWs exhibited outperformance. Such negative response from the high performers can be explained by their frustration of not winning the award despite their higher chances of winning, or emotions such as envy, and jealousy as suggested in several studies (e.g. Salovey and Rodin, 1984; Pelham and Wachsmuth, 1995; Carnahan *et al.*, 2010; Charness *et al.*, 2014; Gallus and Frey, 2017).

Besides the factors related to award design, and individual differences between CHWs, the effectiveness of an award also depends on organizational culture. Literatures in organization studies and labour psychology suggest that compensation and reward systems need to be congruent with management systems and the culture of the organization to have a positive effect on workers’ performance (Morgenstern, 1995; Lundby *et al.*, 1999). For example, a competitive reward system might backfire in organizations that promote a culture of participation and teamwork within the workplace, since such reward systems contradict the espoused values of the organizations (Cameron, 1985). Competitive reward systems would be more congruent with organizational cultures where competitiveness and goal achievement are valued most. BRAC, like most other non-profit organizations, might have promoted a culture of cohesiveness and teamwork with the organization. Introducing a competitive award system, therefore, might have resulted in rejection and low performance from its high-performing CHWs.

Overall, our results contrast with some of the existing literature on upward social comparison (e.g. Ybema and Buunk, 1995; Collins, 1996; Exline and Lobel, 1997) that suggests that high-performing workers are more likely to react positively when other high performers are awarded and that low-performing workers are more likely to react by reducing their performance because of a perception that the chance of getting the award is low. Our results are more in line with Salovey and Rodin (1984) where empirical evidence found that individuals experience social comparison envy or jealousy when they compare themselves with similar and successful rivals. This also follows Ager *et al.* (2016) where evidence was found that in World War II, low-skilled pilots in the German air force showed significant outperformance when one of their fellow pilots was mentioned on the national radio for his outstanding performance.

## Conclusion

Our study focused on CHWs in Uganda. Although the findings may help shed light on important issues of motivating CHWs in other low-income countries, each country will have its own unique history and policies related to CHWs. Findings from Uganda should be interpreted in light of any country-level differences that may vary between low-income countries. Non-random allocation of the awards to branch offices and CHWs is a major shortcoming of our study, which restricts our ability to claim any causal relation between social award and CHW performance. However, our study made use of a unique and large sample of 4050 CHWs with 21 months of observational data. This study, within its limitations, offers two important contributions to the existing literature. First, we contribute to the scarce literature on the effects of non-financial incentives to keep CHWs motivated in low-income settings. Second, we contribute to the literature on the use of awards and the potential for upward social comparisons in the workplace. Our results suggest that best-performer awards can be used as an effective social incentive in boosting up the performance of low-performing CHWs in low-income countries. We also propose that the overall effectiveness of awards can be improved by investing more efforts in, and increasing the transparency of, the nominee selection process.

## Supplementary data

Supplementary data are available at *Health Policy and Planning* online.

*Conflict of interest statement*. Reajul Chowdhury worked for BRAC South Sudan from 2012 to 2016, before the start of the current research. Kevin McKague and Heather Krause declare that they have no conflict of interest..

*Ethical approval*. We received IRB approval from two institutions: The Uganda National Council for Science and Technology (number ARC 186) and the Research Ethics Board of Cape Breton University, Canada (number 1718067).

## Supplementary Material

czaa162_Supp

## Endnotes

1

$\frac{{\sigma _{v1}^2}}{{\sigma _v^2 + {\rm{}}\sigma _{v1}^2 + \sigma _u^2 + {\rm{}}\sigma _e^2}} = \frac{{0.686}}{{0.184 + 0.686 + 0.091 + 0.782}} = 0.39$
