# Supplementary material for: How workers respond to social rewards: evidence from community health workers in Uganda
Source: Health Policy Plan. 2020 Nov 18;36(3):239–48. doi: 10.1093/heapol/czaa162 (PMC8058949; doi:10.1093/heapol/czaa162)
Supplement: czaa162_Supp [file czaa162_supp.zip › Table S1.docx]

**Table S1. Distribution of award-winning CHWs and branches.**

| **Award Giving Month** | **Number of Award Winner CHWs** | **Number of Branches with at Least One Award Winner** |
| --- | --- | --- |
| Dec. 2015 | 46  (1.14%) | 45  (34%) |
| Dec. 2016 | 43  (1.06%) | 43  (32%) |
| Dec. 2017 | 46  (1.14%) | 46  (34%) |
|  | **CHWs** | **Branches** |
| Never received award | 3,915  (97 %) | 46  (34%) |
| Received award once | 135  (3%) | 48  (36%) |
| Received award twice | 0 | 34  (25%) |
| Received award thrice | 0 | 6  (4%) |
