# Supplementary material for: How workers respond to social rewards: evidence from community health workers in Uganda
Source: Health Policy Plan. 2020 Nov 18;36(3):239–48. doi: 10.1093/heapol/czaa162 (PMC8058949; doi:10.1093/heapol/czaa162)
Supplement: czaa162_Supp [file czaa162_supp.zip › Table S2.docx]

**Table S2. Factor loadings of variables**

| **Variables** | **Factor 1** | **Factor 2** |
| --- | --- | --- |
| Log number of families registered | 0.6357 | -0.1184 |
| Log number of pregnancies registered | 0.8482 | -0.0685 |
| Log number of families surveyed | 0.8324 | -0.0875 |
| Log on-time follow-up visits made | 0.7314 | 0.0438 |
| Log number of months (out of 21) CHW was active | 0.2511 | -0.1318 |
| Log_U-1 children assessed | 0.8875 | -0.0982 |
| Log_U-5 children assessed | 0.9374 | -0.1876 |
| Log_U-1 children treated | 0.8452 | -0.0744 |
| Log_U-5 children treated | 0.9407 | -0.1838 |
| Log total health postnatal care visits made | 0.6392 | 0.6936 |
| Log on-time postnatal care visits made | 0.5621 | 0.7668 |
| Log malaria patients of all ages treated | 0.9284 | -0.1812 |

Note: Coefﬁcients >0.3 ensures signiﬁcance at the 0.01 level. Eigen value for Factor 1=7.27. Kaiser–Meyer–Olkin=0.8911 (conﬁrms the model appropriateness). Factor-1 alone explains 61% variance in data.
