# Supplementary material for: How workers respond to social rewards: evidence from community health workers in Uganda
Source: Health Policy Plan. 2020 Nov 18;36(3):239–48. doi: 10.1093/heapol/czaa162 (PMC8058949; doi:10.1093/heapol/czaa162)
Supplement: czaa162_Supp [file czaa162_supp.zip › Table S3.docx]

**Table S3. Effects on CHW peers from the same branch.**

|  | **Random Intercept at Branch Level**  **(1)** | **Random Intercept at CHW Level**  **(2)** | **Random Intercept at Both Levels**  **(3)** | **Random Intercept and Slope at Branch Level and Random Intercept at CHW Level**  **(4)** | **Random Intercept and Slope at Both Levels**  **(5)** |
| --- | --- | --- | --- | --- | --- |
| Treatment: award-winning colleague | -0.023  [-0.041 -0.005] | -0.022  [-0.038 -0.006] | -0.023  [-0.041 -0.006] | -0.049  [-0.225 0.126] | -0.049  [-0.225 0.126] |
|  | (0.009)^**^ | (0.008)^***^ | (0.008)^**^ | (0.089) | (0.089) |
| Branch size | 0.002  [-0.001 0.005] | 0.002  [0.001 0.003] | 0.002  [-0.001 0.005] | 0.001  [-0.004 0.006] | 0.001  [-0.004 0.006] |
|  | (0.002) | (0.000)^***^ | (0.002) | (0.003) | (0.003) |
| Cons | -0.077  [-0.178 0.024] | -0.076  [-0.107 -0.045] | -0.077  [-0.178 0.024] | -0.089  [-0.258 0.079] | -0.089  [-0.258 0.079] |
|  | (0.052) | (0.016)^***^ | (0.051) | (0.086) | (0.086) |
| ICC |  |  |  |  |  |
| Branch | .067  [0.054 0.085] |  | 0.064  [0.050 0.082] | 0.174  [0.141 0.214] | 0.174  [0.141 0.214] |
| CHWs within Branch |  | 0.146  [0.139 0.154] | .151  [0.137 0.167] | 0.259  [0.228 0.294] | 0.259  [0.228 0.294] |
| Additional information: |  |  |  |  |  |
| N | 85,050 | 85,050 | 85,050 | 85,050 | 85,050 |
| LR test: Compared with pooled OLS (chi) | 4,925.55^***^ | 7,291.98*** | 68,465.73^***^ | 14843.51^***^ | 14,843.51^***^ |
| LR test: Other comparison | 1 v. 3: 3540.18^***^ | 2 v. 3: chi = 1173.75^***^ | | 4 v 5: 0 | |
|  |  |  |  |  |  |

Note: Performance index score is the dependent variable. Standard errors in parentheses. 95% confidence intervals are shown in brackets, and standard errors are shows in parentheses. *, **, *** denote statistical significance of coefficients at the 10%, 5%, and 1% confidence level. While each of these multilevel models outperforms the general OLS model in fitting the data, we choose the model in column 4 as our preferred model because it controls for unobserved heterogeneity at both the CHW and branch levels. Furthermore, this model identifies that the spillover effect of the award varies significantly across branch offices. As part of our model identification exercise, we also fitted a model that let the intercept and slope vary at both the CHW and branch levels (see column 5). The likelihood ratio test comparing this model with our preferred model shows that letting the slope vary across CHWs does not add much value in explaining the variance in the data. The χ2 associated with the likelihood ratio test comparing the models is effectively 0.
