# Supplementary material for: How workers respond to social rewards: evidence from community health workers in Uganda
Source: Health Policy Plan. 2020 Nov 18;36(3):239–48. doi: 10.1093/heapol/czaa162 (PMC8058949; doi:10.1093/heapol/czaa162)
Supplement: czaa162_Supp [file czaa162_supp.zip › Table S4.docx]

**Table S4. Fixed Effect Model Estimation and Hausman Test**

|  | Robust Fixed Effect Model  (1) | Fixed Effect Model  (2) | Random Effect  (3) |
| --- | --- | --- | --- |
| Treatment: award-winning colleague | -0.031 | -0.031 | -0.030 |
|  | (0.030) | (0.007) | (0.006)^***^ |
| Month fixed effect | Yes | Yes | Yes |
| Constant | -1.144 | -1.144 | -1.144 |
|  | (0.028)^***^ | (0.011)^***^ | (0.012)^***^ |
| N | 82,215 | 82,215 | 82,215 |
| F | 198.29 | 3761.78 |  |
| *R*^2^ | 0.41 | 0.41 | 0.41 |
| Hoffman Test statistics |  |  |  |
| Chi^2^ value |  | 0.51 | |
| Prob > Chi^2^ |  | 1.00 | |

Note: In estimating the fixed effect model in Column (1), the standard errors have been clustered at branch level. The models in Column (2) and Column (3) are estimated without robust standard errors. The Chi^2^ value associated with the Hausman test is 0.51 (p>Chi2 =1.0) indicating non-correlation between the covariates and the error terms, i.e. the random effect assumption is satisfied.
